# Reply to Saleh et al. COVID-19 and Atherosclerosis: Is There an Association? Comment on “Bielecka et al. From Systemic Inflammation to Vascular Remodeling: Investigating Carotid IMT in COVID-19 Survivors. *Viruses* 2025, *17*, 1196”

**DOI:** 10.3390/v18020190

**Published:** 2026-01-30

**Authors:** Emilia Bielecka-Richter, Piotr Sielatycki, Paulina Pietraszko, Sara Anna Frankowska, Edyta Zbroch

**Affiliations:** Department of Internal Medicine and Hypertension, Medical University of Bialystok, 15-089 Bialystok, Poland; piotr.sielatycki@umb.edu.pl (P.S.); pietraszko270@gmail.com (P.P.); sarafrankowsk@gmail.com (S.A.F.); edyta.zbroch@umb.edu.pl (E.Z.)

We thank the authors for their thorough and constructive comments [1] on our manuscript “From Systemic Inflammation to Vascular Remodeling: Investigating Carotid IMT in COVID-19 Survivors” [2]. We appreciate the opportunity to clarify methodological aspects and fully address all points raised regarding our study design and execution.

Regarding the measurement location and the asymmetrical distribution of atherosclerosis, we would like to clarify that carotid intima-media thickness (CIMT) was measured in the distal segment of the common carotid artery on the far wall, approximately 10 mm proximal to the carotid bulb. This was performed in accordance with the recommendations of the Polish Society of Vascular Surgery. While we acknowledge that this approach evaluates a specific carotid segment, this site is widely recognized for its high reproducibility and is commonly used as a standard reference location in CIMT assessment. Since the aim of our study was to evaluate the progression of subclinical atherosclerosis rather than to quantify total plaque burden, this standardized region provided a reproducible and clinically accepted measurement point.

We also appreciate the important methodological remark regarding cardiac cycle synchronization. Our study did not employ formal synchronization of CIMT measurements with the end-diastolic phase of the cardiac cycle, which constitutes a limitation. To minimize cardiac cycle–related variability, all measurements were performed using a standardized acquisition protocol that included three independent measurements obtained on each side and the use of mean IMT values to reduce random variability. Consistent imaging settings and techniques were applied across all participants. Importantly, previous large-cohort studies demonstrate that cardiac cycle–related fluctuations in CIMT typically range from 0.03 to 0.04 mm, which corresponds well with the variability reported by Polak et al. in the multi-ethnic study of atherosclerosis and in the Framingham offspring cohort [3,4]. This degree of fluctuation is smaller than the inter-individual variability observed in our cohort, making a substantial systematic bias unlikely. Nevertheless, we agree that incorporating automatic cardiac gating would further improve methodological precision in future studies.

Concerning the reproducibility of the baseline location during follow-up, all IMT measurements were performed strictly in accordance with the guidelines of the Polish Society of Vascular Surgery. Following these guidelines, IMT was assessed 10 mm proximal to the carotid bulb, selecting the thickest segment within this predefined region to reduce measurement noise. The reported value represented the mean of repeated measurements to enhance precision. To ensure reproducibility, all follow-up scans were obtained by the same sonographer, using identical anatomical landmarks and referencing saved baseline images for consistent probe positioning. Although 3D mapping was not applied, this standardized approach reduces positional variability and supports reliable comparison between baseline and follow-up measurements.

Regarding intra-reader variability, all measurements were performed by a single sonographer using a consistent and standardized protocol, reducing potential variability arising from inter-observer differences. We acknowledge that a formal assessment of intra-reader variability was not conducted, which represents a methodological limitation. We appreciate this valuable remark and plan to include dedicated repeatability analyses (e.g., Bland–Altman or intraclass correlation coefficient) in future research.

In response to the comment on CIMT pathological cut-off values, we note that our study aimed to evaluate IMT progression over time rather than classify patients based on absolute thresholds; therefore, we did not apply specific pathological cut-off values. Given the variability of normative IMT values across populations and methodological standards, we considered progression to be the most robust parameter in this context. However, referencing commonly used age-adjusted percentiles may provide useful context, which we acknowledge.

Finally, regarding the blinding of the sonographer, we clarify that the sonographer was not formally blinded to participants’ COVID-19 status or measurement time point due to practical clinical constraints. However, the sonographer did not have access to laboratory data (e.g., CRP, lipid ratios), which were central to the primary findings. We agree that full blinding would further minimize potential bias and intend to implement this in future studies.

We once again thank the reviewers for their insightful remarks, which have enabled us to clarify key methodological aspects and highlight the limitations of CIMT as a surrogate marker of subclinical atherosclerosis.
